# Endothelial progenitor cells improve the therapeutic effect of mesenchymal stem cell sheets on irradiated bone defect repair in a rat model

**DOI:** 10.1186/s12967-018-1517-4

**Published:** 2018-05-22

**Authors:** Huan Liu, Yang Jiao, Wei Zhou, Shizhu Bai, Zhihong Feng, Yan Dong, Qian Liu, Xiaoke Feng, Yimin Zhao

**Affiliations:** 1 State Key Laboratory of Military Stomatology & National Clinical Research Center for Oral Diseases & Shaanxi Key Laboratory of Stomatology, Department of Prosthodontics, School of Stomatology, The Fourth Military Medical University, Xi’an, China; 2 Department of Stomatology, PLA Army General Hospital, Beijing, 100700 China

**Keywords:** Endothelial progenitor cell, Bone marrow mesenchymal stem cell, Cell sheet engineering, Bone regeneration, Radiotherapy

## Abstract

**Background:**

The reconstruction of bone defects is often impaired by radiotherapy since bone quality is compromised by radiation. This study aims to investigate the therapeutic efficacy of the composite cell sheets-bone marrow mesenchymal stem cell (BMSC) sheets cocultured with endothelial progenitor cells (EPCs)-in the healing of irradiated bone defects and the biological effects of EPCs on the osteogenic properties of BMSC sheets.

**Methods:**

BMSCs and EPCs were isolated from rat bone marrow. BMSCs were used to form cell sheets by the vitamin C inducing method. EPCs were seeded on BMSC sheets to make EPCs–BMSC sheets. Osteogenesis of EPCs–BMSC sheets and BMSC sheets were tested. In vitro osteogenesis tests included ALP, Alizarin Red S, Sirius Red staining, qRT-PCR and Western blot analysis after 3 and 7 days of osteogenic incubation. Subcutaneous osteogenesis was tested by H&E staining and immunohistochemical staining 8 weeks after transplantation. EPCs–BMSC sheets and BMSC sheets were used in the 3 mm defects of non-irradiated and irradiated rat tibias. Micro-CT and histological analysis were used to test the healing of bone defects 4 and 8 weeks after transplantation.

**Results:**

EPCs–BMSC sheets showed enhanced osteogenic differentiation in vitro with increased expression of osteoblastic markers and osteogenesis related staining compared with BMSC sheets. In subcutaneous osteogenesis test, EPCs–BMSC sheets formed larger areas of new bone and blood vessels. The EPCs–BMSC group had the highest volume of newly formed bone in the defect area of irradiated tibias.

**Conclusions:**

EPCs improved the osteogenic differentiation of BMSC Sheets and enhanced the ectopic bone formation. EPCs–BMSC sheets promoted bone healing in irradiated rat tibias. EPCs–BMSC sheets are potentially useful in the reconstruction of bone defect after radiotherapy.

**Electronic supplementary material:**

The online version of this article (10.1186/s12967-018-1517-4) contains supplementary material, which is available to authorized users.

## Background

The treatment of malignant tumors of bone or adjacent soft tissues often requires surgical procedures and radiotherapy [1]. Surgical bone excision will cause bone defect for the patient and result in aesthetic and functional difficulties. Autologous bone grafts, allografts and synthetic grafting materials are often used for reconstruction of bony structures in clinical practice [2]. However, patients with radiotherapy may have higher rates of flap loss, flap bed–related complication [3] and implant failure [4] since healing of trauma is compromised in irradiated bone [5].

Radiation impairs bone healing due to a complex cascade of cellular and tissue events. The well accepted ‘three-H concept’ holds that radiation leads to hypoxic–hypocellular–hypovascular tissue, tissue breakdown and chronic non-healing wounds [6]. Besides, radiation damages bone marrow microenvironment for stem cells [7]. The number of osteocytes, osteoblasts or osteoclasts is decreased and differentiation of the surviving mesenchymal stem cells (MSCs) and osteoprecursors is inhibited [8].

MSC-based treatment enhances endogenous repair and has long been used for bone defect repair [9]. Researchers used isolated MSC suspensions in combination with biomaterials to improve irradiated bone repair [10, 11]. Although this conventional tissue engineering procedure is widely used in bone tissue regeneration, cell sheet engineering provides an alternative way [12]. The traditional method harvests cells by trypsin digestion, which results in the loss of large numbers of cells and a reduction in cell activity [13]. Cell sheets engineering harvests cells as intact sheets either with a cell scraper [14] or temperature-responsive culture plate [15], thus allowing increased cell numbers and long-term viability [16]. Besides, it preserves cell surface proteins, extracellular matrix, mechanical, chemical, and biological microenvironments [17]. These properties are necessary to re-create functional tissues. Moreover, cell sheets engineering is a beneficial way of cell transplantation as cell sheets can be transplanted and attach to bio-scaffold or directly into the defect area. It overcomes the difficulty of insufficient cell migration and retention on biomaterials. Researchers have shown that bone marrow mesenchymal stem cell (BMSC) sheets can be used to improve bone regeneration [18, 19].

Some researchers tried to make prevascularized MSC sheets by seeding endothelial cells on them to enhance vascularization after implantation [20]. Endothelial progenitor cells (EPCs) are attractive candidates to address vascular issues [21] and have been found to participate in vessel regeneration after brain radiation in mice [22]. It is of interest to test whether EPCs could be used in combination with BMSC sheets to improve bone healing in irradiated bone.

The goal of this study was to test the therapeutic efficacy of EPCs–BMSC sheets in irradiated bone defects repair. BMSCs were used to form cell sheets and EPCs were seeded on BMSC sheets to make EPCs–BMSC sheets. We assessed the in vitro osteogenic differentiation and subcutaneous osteogenesis of EPCs–BMSC sheets and BMSC sheets. We evaluated the healing of bone defects after implanting cell sheets. The result suggests that EPCs–BMSC sheets are potentially useful to improve the regeneration of irradiated bone.

## Methods

### Cell isolation, cultivation, and characterization

The isolation and primary culture procedure of rat BMSCs and EPCs have been previously reported [23]. Bone marrow was flushed from tibias and femurs of rats. The bone marrow suspension was fractionated by density gradient centrifugation (Histopaque-1083, Sigma, USA) for 25 min at 400*g* and the mononuclear cells were used. BMSCs were cultured in α-minimum essential medium (α-MEM, Gibco, USA) supplemented with 10% fetal bovine serum (FBS, Hangzhou Sijiqing Biological Engineering Materials Co., Ltd. China) and 1% penicillin and streptomycin. Cells of the third passage were tested for osteogenic, adipogenic and chondrogenic differentiation and cell surface markers. EPCs were suspended in EBM-2 medium with EGM-2 MV SingleQuots (Lonza, USA). The non-adherent cells were transferred to new dishes after 48 h. EPCs of the third passage were tested for cell surface markers, capillary tube formation, Weible–Palade bodies and uptake of Dil-Ac-LDL and FITC-UEA-1.

### Cell sheets preparation

BMSCs of the third passage were seeded in 6-well plates at the density of 3 × 10^5^ cells/well. The medium was shifted to cell sheet-inducing medium (α-MEM supplemented with 10% FBS, 50 µg/ml Vc and 1% penicillin and streptomycin) after cells reached 95% confluence. Cell sheets were formed after 8 days of culture. EPCs (2 × 10^5^) were seeded onto BMSC sheets to make EPCs–BMSC sheets (+EPC). The composite sheets were cultured for 48 h to ensure EPCs’ adherence. BMSC sheets (BMSC) without EPCs suspension were further cultured in cell sheet-inducing medium for 48 h.

### Structural observation of cell sheets

To observe EPCs’ adherence to BMSC sheets, long-chain carbocyanine membrane probes DiL and DiO were used to label BMSCs and EPCs. 1 × 10^6^ BMSCs were suspended with 1 ml serum-free medium. 5 μl DiL (1 mM) were added to the cell suspension. After incubation for 5 min at 37 °C and 15 min at 4 °C, cells were washed with PBS and used for cell sheet preparation. EPCs were labeled with DiO, and the labeling protocol was the same as DiL. The DiO labeled EPCs were seeded onto BMSC sheets. After incubation for 48 h, cells were observed with an inverted fluorescence microscope (Leica DMI6000B).

Cell sheets were fixed with 4% paraformaldehyde, embedded in paraffin and cut into 5-μm thick sections for the H&E staining. For SEM observation, cell sheets were dehydrated and coated with gold and examined by a scanning electron microscope (SEM, Hitachi S-4800).

### In vitro osteogenic differentiation of cell sheets

Cell sheets of BMSC group and +EPC group were incubated with osteogenic medium (10 mM β-glycerolphosphate, 50 µg/ml Vc and 0.1 mM dexamethasone, Sigma–Aldrich) for 3 or 7 days. ALP production was tested by BCIP/NBT ALP color development kit (Beyotime, China). ALP activity was tested by ALP assay kit (Nanjing Jiancheng Bioengineering Institute, China). Extra cellular matrix (ECM) mineralized nodules were stained with 1 wt% Alizarin Red S (Beyotime, China). The stain was dissolved in 10% cetylpyridinium chloride in 10 mM sodium phosphate and the absorbance was measured at 620 nm for quantification. Collagen secretion was stained with Sirius Red (Leagene, China). The stain was dissolved in the destain solution (0.2 M NaOH/methanol 1:1), and the absorbance was measured at 540 nm for quantification.

Quantitative real-time polymerase chain reaction (qRT-PCR) was used to detect the gene expression of *Runx2*, *Alp*, *Bmp2*, *Ocn* and *Vegf*. Briefly, total RNA was extracted using TriZol (Invitrogen, USA) and 500 ng total RNA was transcribed into cDNA using a PrimeScript RT reagent kit (TaKaRa, Japan). The analysis was performed on the CFX96™Real Time RT-PCR System with SYBR PremixExTaq™II (TaKaRa, Japan). The relative gene expression was determined using the ΔΔCt method. The primers were synthesized as shown in Table 1.

**Table1:** Primers used for qRT-PCR

Gene	Forward primer sequence (5′–3′)	Reverse primer sequence (5′–3′)
*Runx2*	5′ AGA CCA GCA GCA CTC CAT AT 3′	5′ CTC ATC CAT TCT GCC GCT AGA 3′
*Alp*	5′ ATG GCT CAC CTG CTT CAC G 3′	5′ TCA GAA CAG GGT GCG TAG G 3′
*Bmp2*	5′ ATG GGT TTG TGG TGG AAG TG 3′	5′ TTG GCT TGA CGC TTT TCT CG 3′
*Vegf*	5′ AGG AGT ACC CCG ATG AGA TA 3′	5′ CTT CTA CTG CCC TCC TTG TA 3′
Ocn	5′ AGG GCA GTA AGG TGG TGA AT 3′	5′ GCA TTA ACC AAC ACG GGG TA 3′
*Gapdh*	5′ GGCACAGTCAAGGCTGAGAATG3	5′ ATGGTGGTGAAGACGCCAGTA3′

Protein expression of Runx2, ALP, BMP-2, OCN, VEGF and GAPDH was detected by Western blot. Cell sheets were lysed in RIPA buffer with a protease inhibitor cocktail (Sigma, USA). Protein concentrations were quantified by the BCA protein assay (Beyotime, China). Proteins were separated by SDS-PAGE and transferred to the PVDF membranes. The membranes were blocked with 5% BSA for 2 h and incubated with primary antibodies for rat RUNX2 (Santa Cruz Biotechnology, sc-10758), ALP (Protein tech, 11187-1-AP), BMP2 (Abcam, ab14933), OCN (Abcam, ab13418), VEGF (Abcam, ab46154) and GAPDH (Abcam, ab8245). The membranes were incubated for 2 h with secondary antibodies (Cowin Biotech, China). The protein bands were visualized with a detection system (Amersham Biosciences, USA). The gray values of the protein bands were quantified by using Image-Pro Plus 6.0 software.

### Subcutaneous osteogenesis of cell sheets

Cell sheets of BMSC group and +EPC group were wrapped around titanium implants (99.99% pure; Zhong Bang Corporation, China) and subcutaneously transplanted into the backs of nude mice (n = 3). Samples were harvested and fixed with 4% paraformaldehyde 8 weeks after implantation. Peri-implant tissues were detached from titanium implants and decalcified for 14 days in 5% EDTA (pH 7.0). The specimens were prepared for H&E staining and immunohistochemical staining of BMP-2 (1:200; Abcam), OCN (1:200; Abcam), VEGF (1:200; Abcam), CD31 (1:200; Abcam).

### Bone regeneration in surgically created defects

#### Experimental design

Male Sprague–Dawley rats weighing 240–270 g were used. 15 rats received irradiation for their tibias and 15 rats were not irradiated. Bone defect surgeries were performed 8 weeks after irradiation. Tibias of irradiated rats and non-irradiated rats were randomly allocated into the following groups: (1) +EPC, (2) BMSC, (3) CTR (control) for 4 weeks (n = 4) and 8 weeks (n = 6). Samples were used for micro-CT analysis and histological evaluation.

#### Radiation

Tibias of each rat were irradiated with a single dose of 20 Gy using the 23EX medical linear accelerator (Varian, USA). Radiation was delivered at energy 6 MeV, dose rate 4 Gy/min. Lead shielding was used to protect the rest parts of rats.

#### Bone defect surgeries

An incision of 15 mm long was made on the mesial surface of the tibia. A 3-mm-diameter defect was created in tibial metaphysis. The defect was filled with cell sheets of the +EPC group or BMSC group or empty. Afterwards, muscle tissue and skin were sutured separately.

#### Sequential fluorescent labeling

Different fluorochromes were injected intramuscularly. Alizarin Red S (Sigma, USA, 30 mg/kg) injections were performed at 2 and 3 weeks post-operation. Calcein (Sigma, USA, 20 mg/kg) injections were conducted at 4 and 5 weeks post-operation. Tetracycline Hydrochloride (Sigma, USA, 20 mg/kg) injections were performed at 6 and 7 weeks post-operation.

#### Test of micro-CT

Tibias were harvested and fixed in 4% paraformaldehyde. The samples were scanned by Micro-CT (Y.XLONY.Cheetah, Germany) with the resolution of 13 µm. Three-dimensional (3D) images were reconstructed with VG StudioMAX (Volume Graphics, Germany). The region of interest (ROI) was the original cylindrically shaped bone defect (L: 2 mm; φ: 3 mm). Bone volume/total volume (BV/TV) was calculated.

#### Histological analysis

For H&E staining, samples were decalcified for 21 days in 5% EDTA (pH 7.0) and prepared according to standard protocols.

For hard tissue slices examination, samples were dehydrated with ethanol of ascending concentrations, embedded in polymethylmethacrylate (PMMA) and cut into sections using a microtome (LEICA SP1600, Germany). The fluorescent labeling was observed using the Stereo Microscope and Laser Scanning Confocal Microscope (OLYMPUSFV1000, Japan). The area of three fluorochromes stained bone was quantified by Image-Pro Plus 6.0 software.

#### Statistical analysis

Results were expressed as mean ± standard deviation. Data were analyzed by Student t-test or one way ANOVA followed by Tukey post-test. GraphPad Prism7 software was used and statistical significance was considered when p < 0.05.

## Results

### Characterization of BMSCs and EPCs

BMSCs showed a spindle-shaped morphology, expressed CD29, CD44, CD90 positively and were negative for CD31, CD34. BMSCs exhibited osteogenic, adipogenic and chondrogenic differentiation ability (Additional file 1: Figure S1A).

EPCs showed a cobblestone-like morphology and expressed CD31, CD144, VEGFR2 positively. EPCs exhibited tube-like structure when seeded on Matrigel. Weible–Palade bodies, the endothelial specific organelles, were observed in EPCs under the transmission electron microscope. The cells could uptake Dil-Ac-LDL and bond FITC-CEA-1 (Additional file 1: Figure S1B).

### The structure of cell sheets

**Fig. 1:**
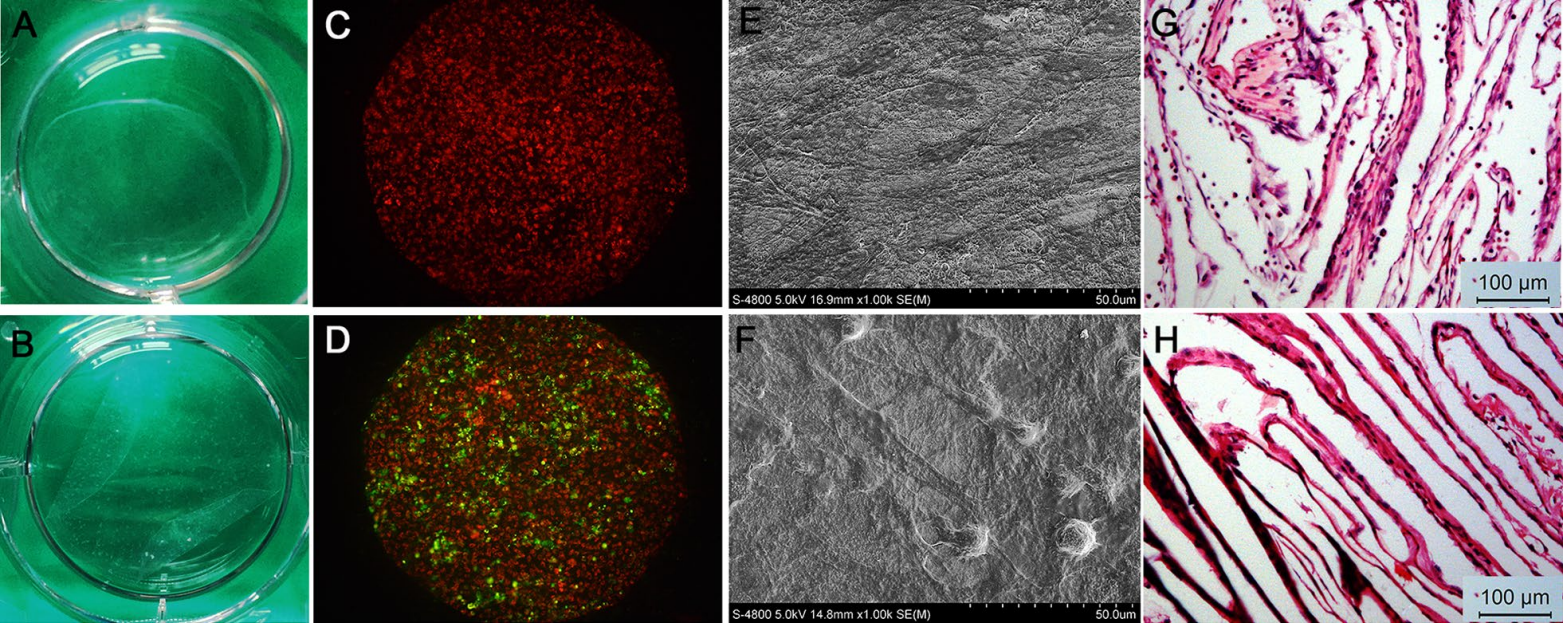
Structure of cell sheets. Macroscopic images of cell sheets of the BMSC group (**A**) and +EPC group (**B**) detached from culture dishes. Representative microscopic view of cell sheet morphology of the BMSC group (**C**) and +EPC group (**D**); BMSCs were stained with Dil (red) and EPCs were stained with DiO (green). Representative SEM images of cell sheets of BMSC group (**E**) and +EPC group (**F**). H&E staining images (×100) of cell sheet of BMSC group (**G**) and +EPC group (**H**)

Cell sheets could be detached from plates (Fig. 1 A, B). The DIO labelled EPCs could adhere and extend on the DIL labelled BMSC sheets (Fig. 1C, D). The SEM examination revealed that the BMSC sheets were composed of a dense cellular network with abundant ECM (Fig. 1E). EPCs were stretched on the BMSC sheets (Fig. 1F). H&E staining revealed that both cell sheets were about 30 ± 5 μm in thickness (Fig. 1G, H).

### In vitro osteogenic differentiation of cell sheets

**Fig. 2:**
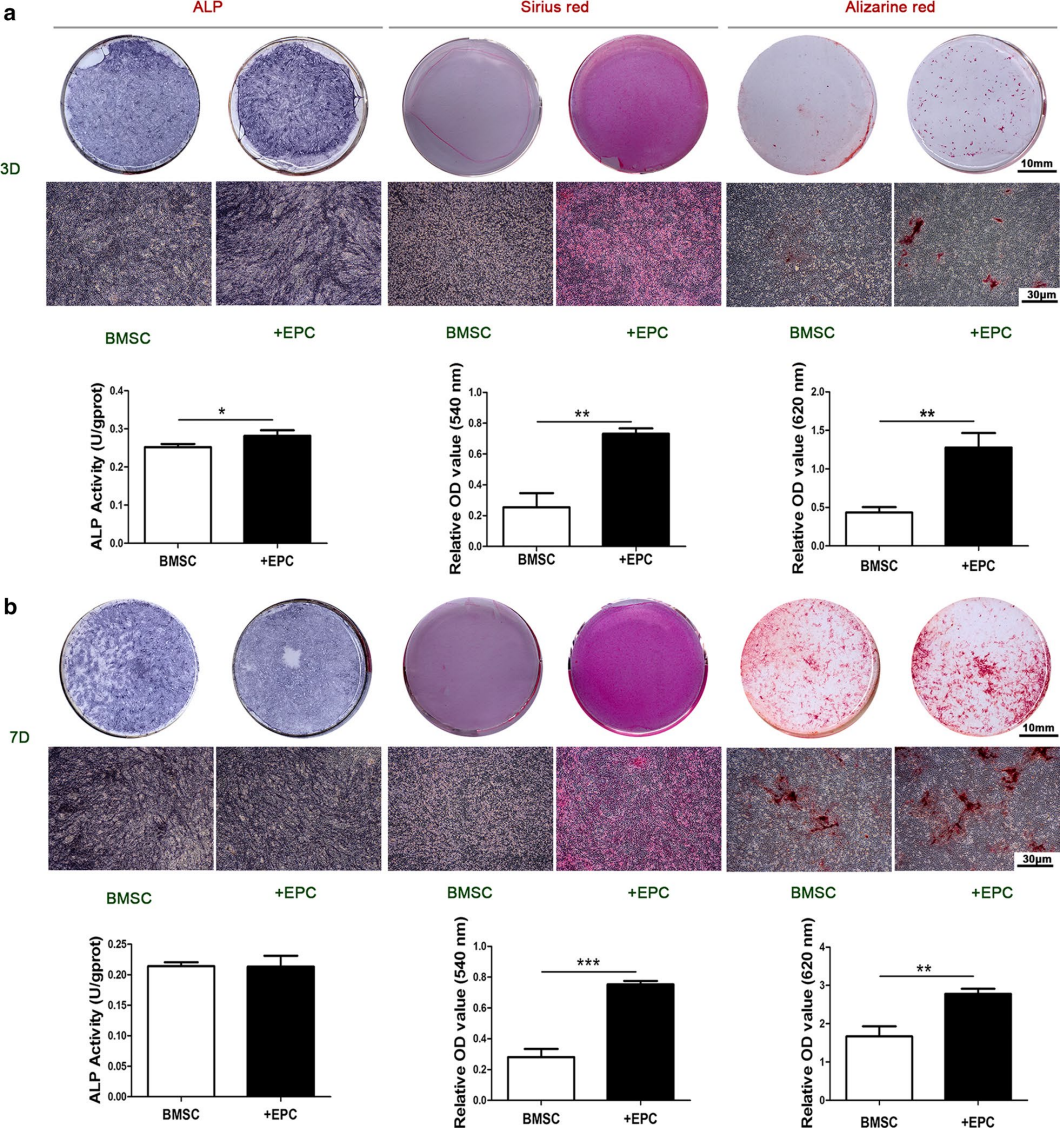
ALP staining, ALP activity, collagen secretion and ECM mineralization of cell sheets after osteogenic differentiation. ALP staining, intracellular ALP activity, collagen secretion, ECM mineralization and the quantitative colorimetric results of cell sheets after 3 (**a**) and 7 days (**b**) of osteogenic induction. Representative macroscopic view is shown in the upper panels and microscopic view (×40) is shown in the lower panels. Data are shown as mean ± SD and analyzed by Student t-test, n = 3; *p < 0.05, **p < 0.01 and ***p < 0.001

After 3 days of osteogenic induction, the production of ALP was higher in the +EPC group than the BMSC group, as indicated by the density of ALP staining and the intracellular ALP activity (p < 0.05). There was no significant difference in ALP staining and ALP activity between the two groups after 7 days of induction (p > 0.05). The secretion of collagen and the ECM mineralization were higher in the +EPC group than the BMSC group both on the 3rd day (p < 0.01) and the 7th day (p < 0.001 or 0.01) of induction (Fig. 2).

**Fig. 3:**
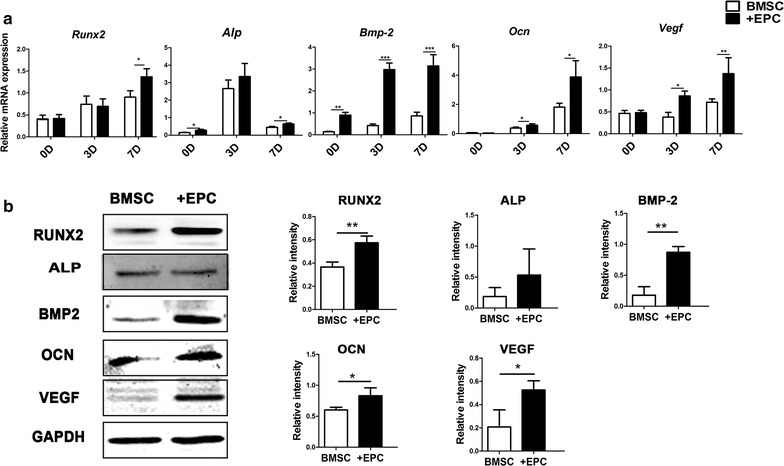
Osteogenic genes and proteins expression of cell sheets after osteogenic differentiation. **a** Gene expression of *Runx2, Alp, Bmp*-*2, Ocn* and *Vegf* after 0, 3 and 7 days of osteogenic induction. **b** Western blot analysis of RUNX2, ALP, BMP-2, OCN, VEGF and GAPDH after 7 days of osteogenic induction and the quantitative analysis of the protein bands. Data are shown as mean ± SD and analyzed by Student t-test, n = 3; *p < 0.05, **p < 0.01 and ***p < 0.001

The gene expression of *Runx2* and *Alp* were higher in the +EPC group than the BMSC group at day 7 (p < 0.05). The +EPC group had higher expression of *Bmp2*, *Ocn* and *Vegf* than the BMSC group at both time points of induction (p < 0.05, 0.01 or 0.001) (Fig. 3a).

There was no significant difference in the protein expression of RUNX2, ALP, OCN and VEGF after 0 and 3 days of osteogenic induction between the two groups (p > 0.05), whereas the expression of BMP2 was higher in the +EPC group (p < 0.001) (Additional file 2: Figure S2). The +EPC group had higher expression of RUNX2, BMP2 OCN and VEGF than the BMSC group after 7 days of osteogenic induction (p < 0.05 or 0.01), whereas no significant difference in ALP protein level was observed (p > 0.05) (Fig. 3b).

### Subcutaneous osteogenesis of cell sheets

**Fig. 4:**
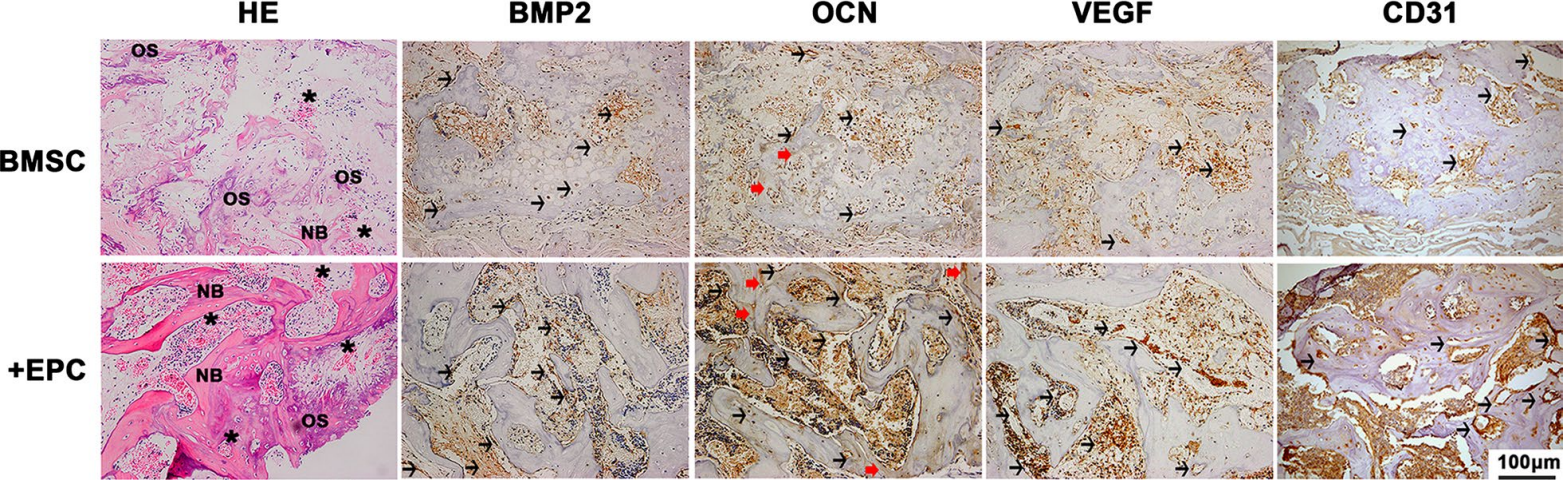
Subcutaneous osteogenesis of cell sheets. Representative H&E (×200) and immunohistochemical staining images (×200) of BMP-2, OCN, VEGF and CD31. New bone formation (NB), osteoid (OS), blood vessels (asterisk) are indicated in the images. Black arrows indicate positive staining cells, red arrows indicate positive staining bone matrix

In the BMSC group, osteoid was mainly detected, which is indicated by slightly darker staining than the mineralized bone. The +EPC group showed continuous bone formation and was rich in blood vessels (Fig. 4). The +EPC group showed increased BMP2 staining in the number of lining cells along new bone than the BMSC group. However, the BMSC group showed higher staining in connective tissue than the +EPC group. Hypertrophic chondrocytes were observed in the BMSC group but not in the +EPC group, and they were also positive for BMP2 staining. OCN staining was located within the cells and newly formed bone, and the +EPC group showed greater staining than the BMSC group. VEGF was detected in osteoblasts and endothelial cells in both groups and the expression was higher in the +EPC group. The number of CD31 positive vessels was increased in the +EPC group compared to the BMSC group.

### The function of cell sheets on bone repair

**Fig. 5:**
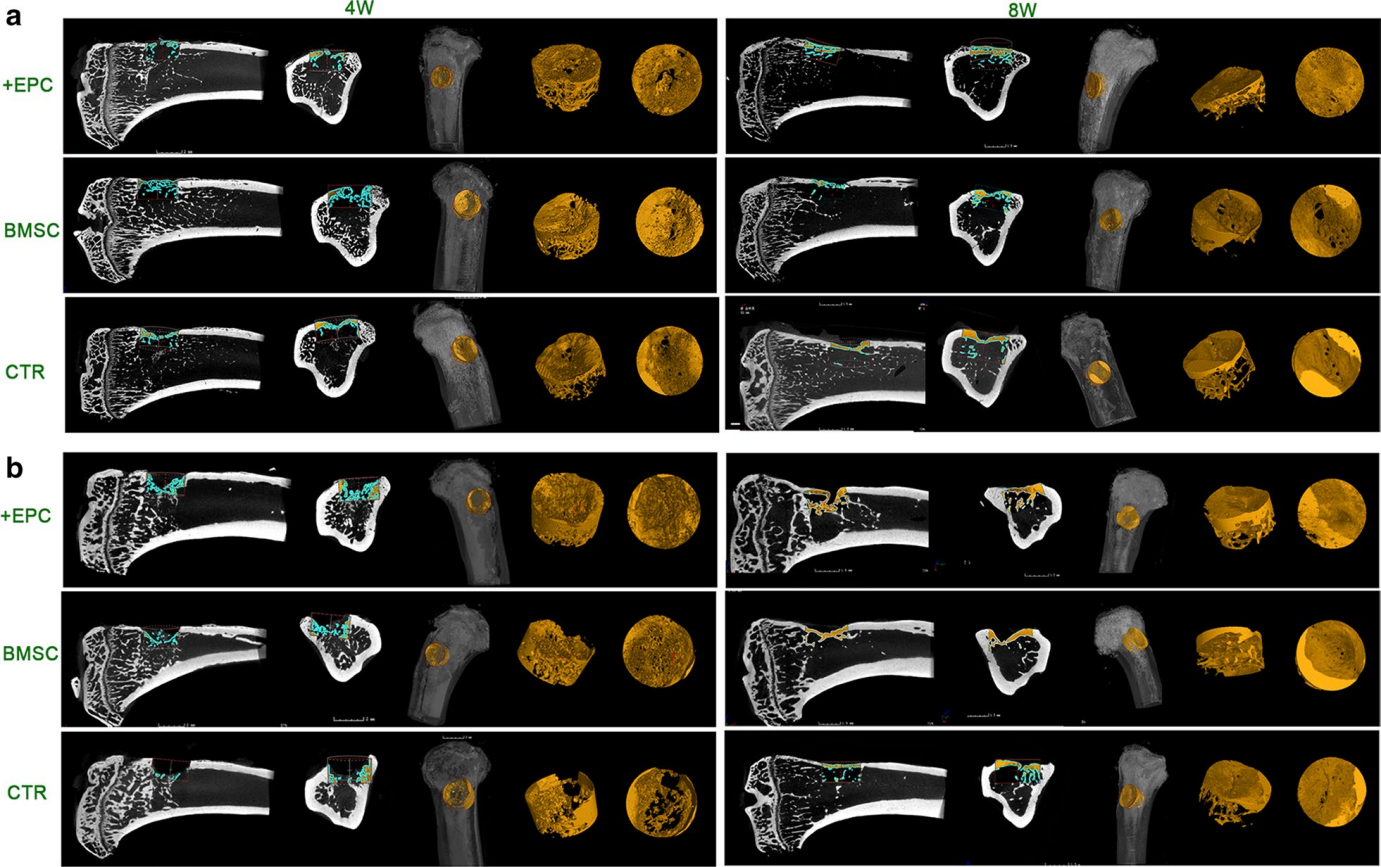
Micro-CT evaluation of newly formed bone. 2D and 3D images of the bone formed in the defect area of non-irradiated (**a**) and irradiated (**b**) rats at 4 and 8 weeks. Bone structure within the ROI is shown in orange in the 3D reconstructed images. The lateral and coronal views of the reconstructed defect area are shown

**Fig. 6:**
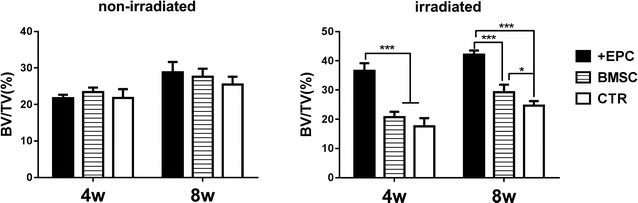
The quantitative results of Micro-CT evaluation. The graphs show the morphometric analysis of the bone volume to total volume ratio (BV/TV). Data are shown as mean ± SD and analyzed by one way ANOVA followed by Tukey post-test, n = 4; *p < 0.05, ***p < 0.001

For those non-irradiated rats (Figs. 5a, 6; Additional file 3: Tables S1, S2), new bone formation was detected in both cortical bone area and trabecular bone area in each group at 4 weeks after surgery. At 8 weeks, newly formed bone tissues almost filled the entire cortical defect area. The bone volume had no significant difference among the three groups (p > 0.05). For those irradiated rats (Fig. 5b, 6; Additional file 3: Tables S1, S2), no new bone formation was detected in the cortical bone area, while trabecular bone healing could be detected 4 weeks after surgery. The volume of newly formed bone in the +EPC group was higher than the other two groups (p < 0.001). At 8 weeks, the volume of newly formed bone in the +EPC group was the highest among the three groups (p < 0.001), and the BMSC group also showed higher BV/TV than the control group (p < 0.05).

**Fig. 7:**
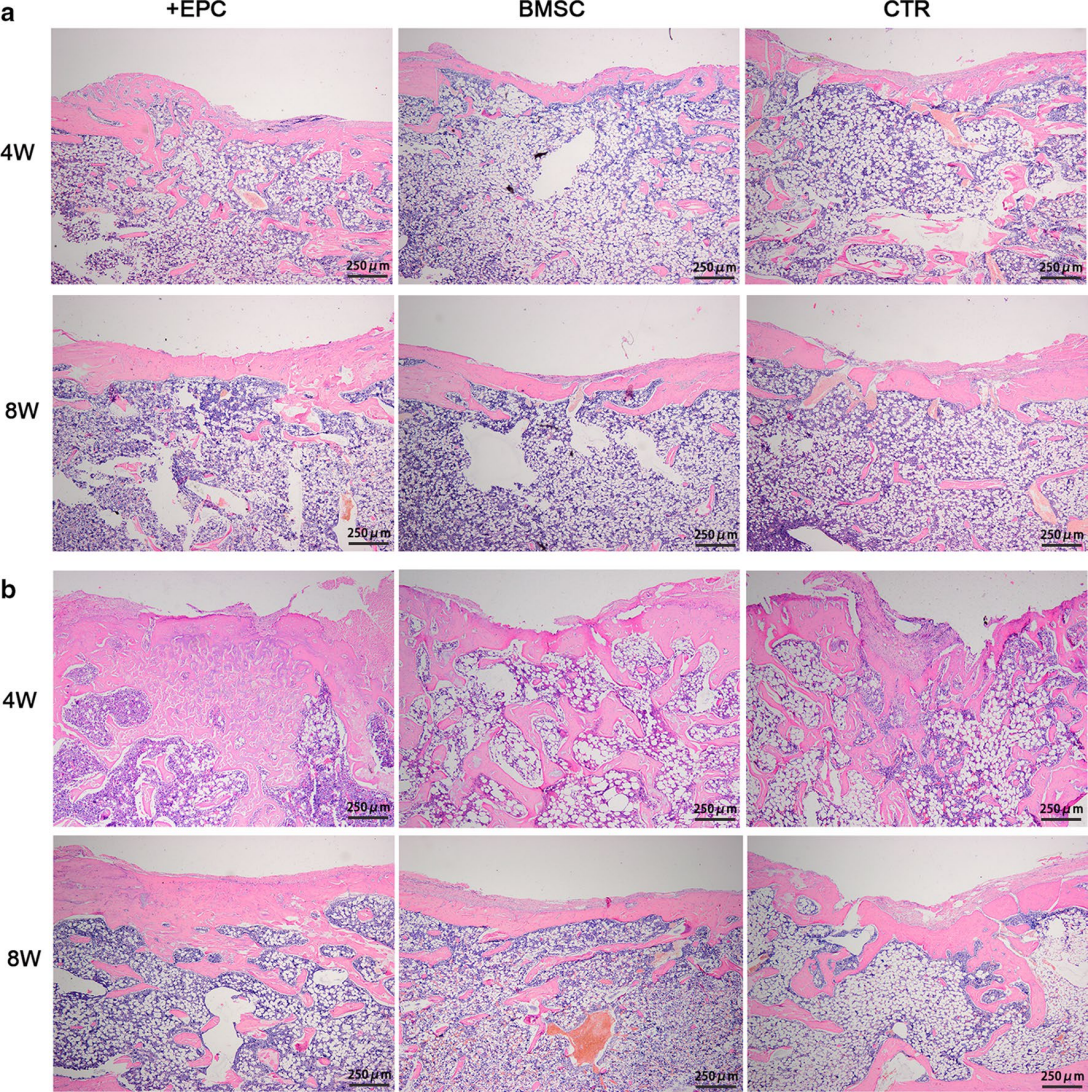
Histological analysis of newly formed bone. H&E staining of tibial defects of the non-irradiated (**a**) and irradiated (**b**) rats at 4 and 8 weeks (×40)

H&E staining was carried out to evaluate the newly formed bone tissue. For those non-irradiated rats (Fig. 7a), thin bony bridge spanned the cortical window at 4 weeks, and trabecular bone was observed in the defect area. At 8 weeks, the cortical defects were entirely bridged by new cortical bone, whereas the trabecular bone in the medullary canal had decreased to a level comparable with the intact bone. No significant difference of new bone formation was observed among the three groups at both time points. For those irradiated rats (Fig. 7b), bone had formed and filled the central region in all groups at 4 weeks. In the +EPC group, the medullary cavity in the defect area was filled with abundant woven new bone, and new cortical bone was observed. In the BMSC group, new cortical bone was formed, but the original bone contour was not achieved. In the control group, the newly formed cortical bone was less than the other two groups and the soft tissue on the periosteal side displayed down-growth into the defect area. At 8 weeks, the cortical defect was completely bridged by new cortical bone, and the trabecular bone in the defect area was decreased in each group.

**Fig. 8:**
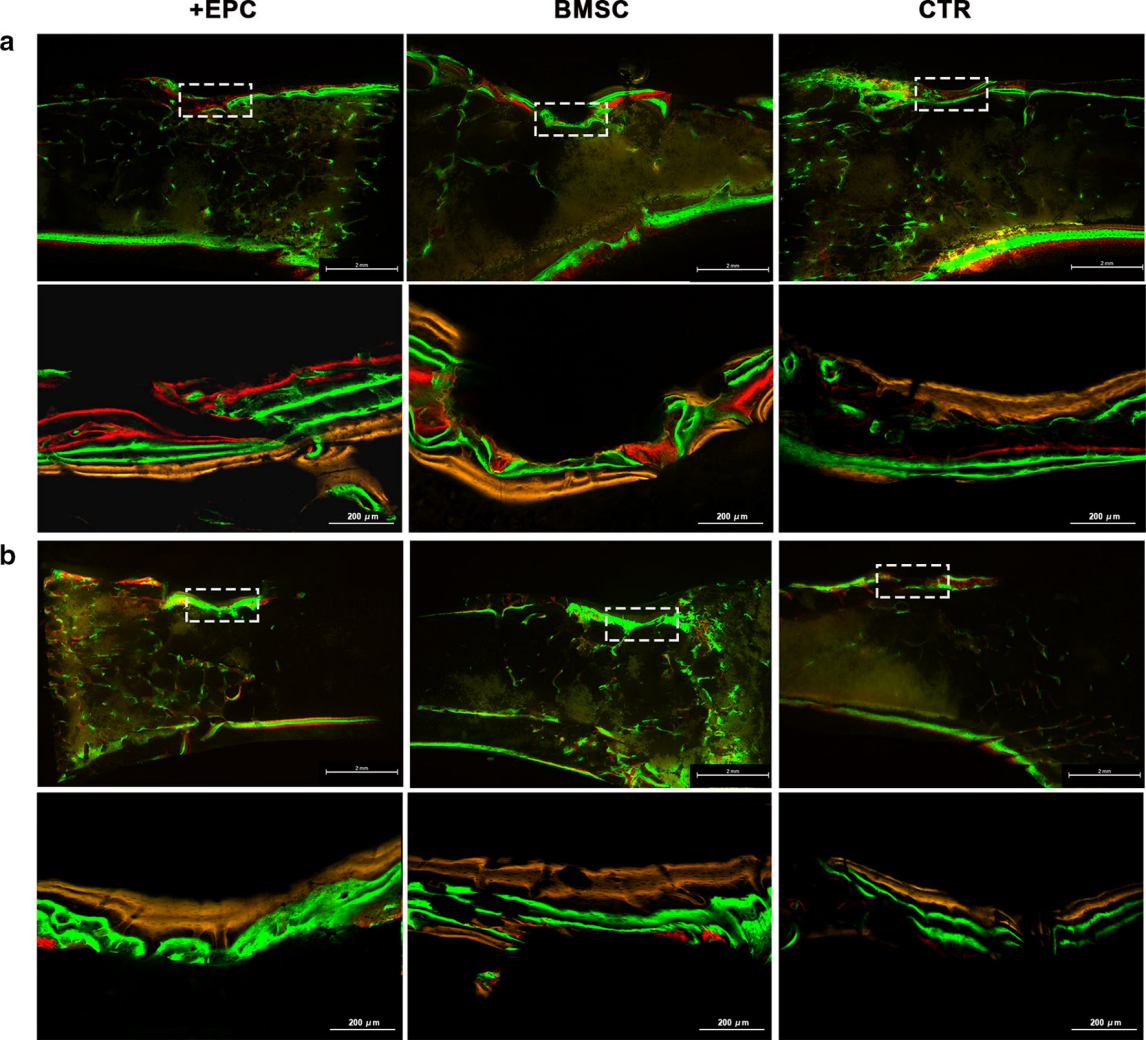
Sequential fluorescent labeling of bone formation and mineralization. Bone formation and mineralization in the defect area of the non-irradiated (**a**) and irradiated (**b**) rats at 8 weeks. Red, green and yellow represent labeling by Alizarin Red S (AL), calcein (CA) and tetracycline hydrochloride (TE). The upper panels show the overall image of each group (×15). The lower panels show the area within the white box and indicate new bone tissues formed in the defect (×100)

**Fig. 9:**
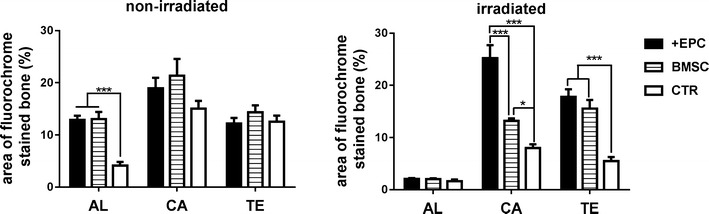
The quantitative results of sequential fluorescent labeling. The graphs show the area of bone stained with Alizarin Red S (AL), calcein (CA) and tetracycline hydrochloride (TE). Data are shown as mean ± SD and analyzed by one way ANOVA followed by Tukey post-test, n = 3; *p < 0.05, ***p < 0.001

Sequential fluorescent labeling was used to measure bone mineralization and deposition (Figs. 8, 9; Additional file 3: Tables S3, S4). For those non-irradiated rats, Alizarin Red S labeling area of the +EPC group and BMSC group was larger than the control group (p < 0.001). For those irradiated rats, little Alizarin Red S labeling area was observed. Calcein labeling area in the +EPC group was larger than the other two groups (p < 0.001). BMSC group also showed larger calcein labeling area than the control group (p < 0.05). Tetracycline Hydrochloride area in the +EPC group and BMSC group was larger than the control group (p < 0.001).

## Discussion

Radiotherapy acts on tumors by killing rapidly dividing malignant cells; however, it also damages healthy cells [24]. Damaging of vascular endothelium cells and osteoblastic cells in irradiated bone inhibits bone healing. This study aims to verify the possibility of using EPCs–BMSC sheets to improve irradiated bone defect repair. Our results demonstrated that EPCs could enhance the osteogenic differentiation in vitro and in vivo. When implanted in bone defects, EPCs–BMSC sheets enhanced the repair of bone defects in irradiated rats.

BMSCs are bone marrow-derived stem cells and first described by Friedenstein [25]. BMSCs are capable of differentiating into osteoblastic, chondrocytic and adipogenic lineages [26]. There is no specific markers for their identification, but CD44, CD29, CD90 and CD105 are commonly used [27–29]. EPCs are initially identified by Isner and Asahara [30]. EPCs are precursor cells of vascular endothelial cells and participate in angiogenesis and neovascularization. EPCs are characterized by the expression of endothelial markers such as VEGFR-2, CD31, CD144 (VE-cadherin), Tie-2, CD133, vWF and the haematopoietic stem cell marker CD34 [31, 32].

Cell sheet engineering technology has been used to make highly vascularized tissues such as corneal, myocardial, hepatic, and periodontal tissues. To enhance neovascularization after transplantation, researchers tried to make cell-sheets with vascular networks in culture [33]. Seeding human umbilical vein endothelial cells (HUVECs) on MSCs cell sheets resulted in prevascularized cell sheets and promoted the formation of blood vessels in vivo [20]. In our study, we seeded EPCs on BMSC sheets. Compared with mature endothelial cells, EPCs have the advantage of higher proliferation rate and survival potential [34]. EPCs could facilitate vessel formation by differentiating into endothelial cells and incorporating into newly formed vessels or producing pro-angiogenic factors [35]. We found that EPCs could adhere and extend on cell sheets, but they didn’t develop vascular structure. When transplanted into nude mice, EPCs–BMSC group showed more blood vessel networks than the BMSC group.

We used irradiated and non-irradiated rats to test whether EPCs–BMSC sheets could improve bone regeneration. Our data showed that EPCs–BMSC sheets improved bone repair of irradiated rats. For those non-irradiated rats, the defects in all groups healed 8 weeks after surgery as rats have quick skeletal changes, bone turnover and very brisk bone healing [36]. But we still observed that EPCs–BMSC sheets and BMSCs sheets enhanced bone healing within the first 2 weeks by the sequential fluorescent labeling test. Other research also revealed that the co-cultured EPCs and BMSCs had enhanced osteogenic differentiation ability compared to BMSCs [37], and co-transplantation of MSCs and EPCs/ECs improved the healing of bone defects [38].

There are some possible mechanisms of EPCs–BMSC sheets in improving the regeneration of irradiated bone. The compromised irradiated bone repair are due to the stem cell depletion [39]. EPCs–BMSC sheets could provide cells of both endothelial and osteoblastic lineages. Besides, the osteogenic growth factors and the cells producing them are lacking in irradiated bone [10]. We demonstrated that EPCs enhanced BMP2 and VEGF expression of BMSC sheets. VEGF and BMP-2 are essential for bone formation and repair as they could stimulate osteogenic differentiation and maturation of MSCs [40]. This could be the reason that EPCs–BMSC sheets performed better than BMSC sheets in our research. Moreover, EPC-mediated neovascularization could facilitate oxygen and nutrition supply for the implanted tissue and contribute to bone regeneration [41]. In the subcutaneous osteogenesis test, in addition to the increased expression of BMP2 and VEGF, enhanced neovascularization was observed. Further information on the mechanisms of EPCs–BMSC sheets in enhancing irradiated bone repair should be explored.

However, some discrepancies exist between the defect model and large clinical defects. We used cell sheets in a gap healing model where mechanically stability remained, but just the use of cell sheet cannot provide the mechanical stability in large defects. The therapeutic potential of EPCs–BMSC sheets in irradiated bone defect could be further explored with combination use of bone grafts, endo-osseous implants and other tissue engineering methods.

## Conclusion

It is a novel approach to enhance defect healing in irradiated bone by cell sheet engineering. Results demonstrated that EPCs improved the osteogenic differentiation and the ectopic bone formation of BMSC Sheets. EPCs–BMSC sheets enhanced bone healing in irradiated rat tibias. Using of EPCs–BMSC sheets in irradiated bone defect could supply cells of the mesenchymal stem cell-osteoblast lineage and vascular lineage. Our findings suggest that EPCs–BMSC sheets have the potential for future use to improve the regeneration of irradiated bone.

## Additional files

**Additional file 1: Figure S1.** Characterization of BMSCs and EPCs.

**Additional file 2: Figure S2.** Protein expression of cell sheets after 0 and 3 days of osteogenic induction.

**Additional file 3: Table S1.** BV/TV (%) of the micro-CT evaluation. **Table S2.** p value of comparison of BV/TV. **Table S3.** Area of fluorochromes stained bone (%). **Table S4.** p value of comparison of fluorochromes stained area.

## Abbreviations

EPC: endothelial progenitor cell
BMSC: bone marrow mesenchymal stem
MSC: mesenchymal stem cell
RUNX2: runt-related transcription factor 2
VEGF: vascular endothelial growth factor
ALP: alkaline phosphatase
BMP-2: bone morphogenetic protein 2
OCN: osteocalcin
GAPDH: glyceraldehyde phosphate dehydrogenase
ECM: extra cellular matrix
H&E: hematoxylin and eosin
qRT-PCR: quantitative real-time polymerase chain reaction
