# Supplementary figures and images for: Endothelial progenitor cells improve the therapeutic effect of mesenchymal stem cell sheets on irradiated bone defect repair in a rat model
Source: J Transl Med. 2018 May 22;16:137. doi: 10.1186/s12967-018-1517-4 (PMC5964689; doi:10.1186/s12967-018-1517-4)

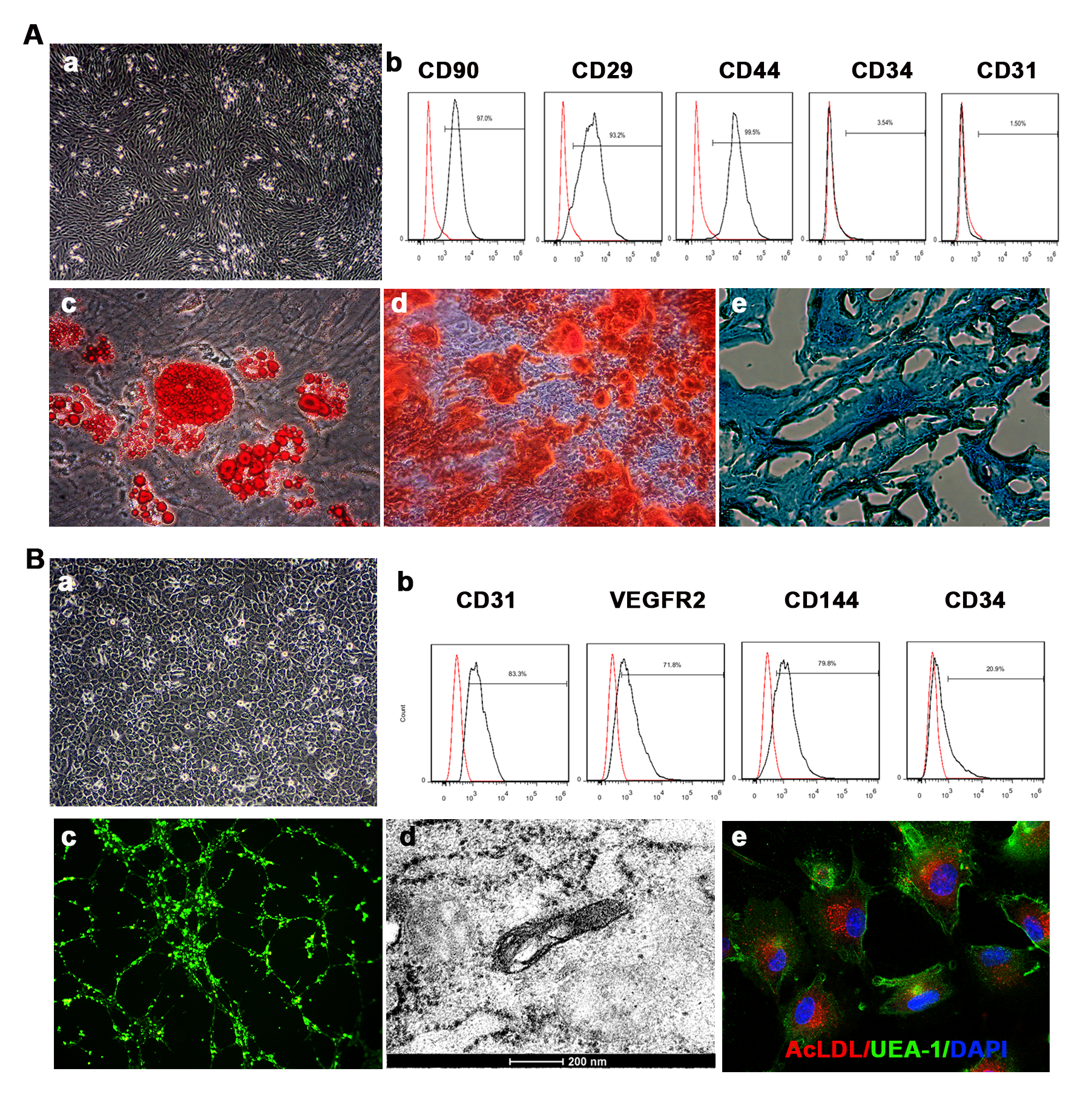

Supplement: Supplementary file 1 — Additional file 1: Figure S1. Characterization of BMSCs and EPCs. [file 12967_2018_1517_MOESM1_ESM.tif]

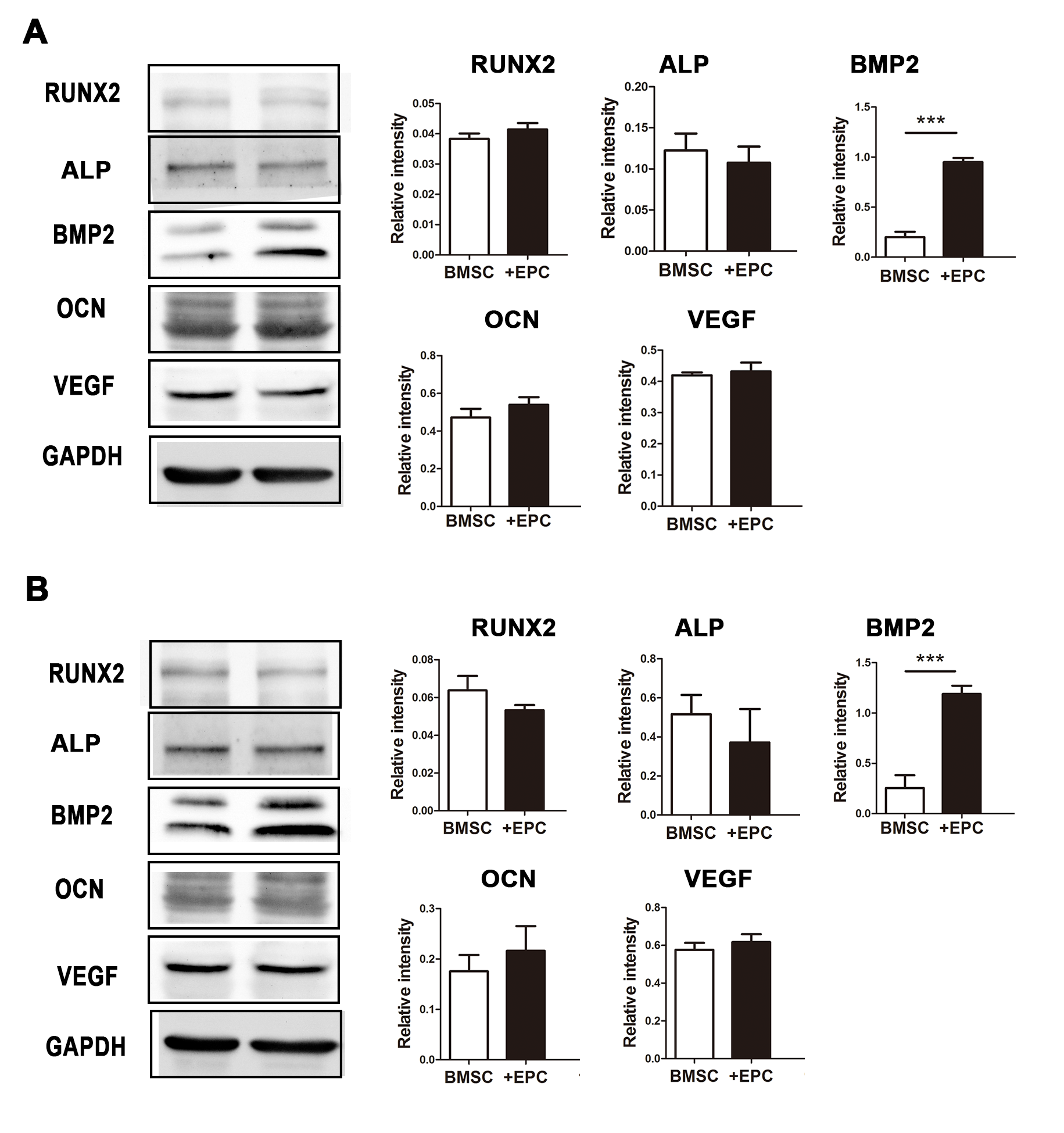

Supplement: Supplementary file 2 — Additional file 2: Figure S2. Protein expression of cell sheets after 0 and 3 days of osteogenic induction. [file 12967_2018_1517_MOESM2_ESM.tif]
