# Supplementary material for: Endothelial progenitor cells improve the therapeutic effect of mesenchymal stem cell sheets on irradiated bone defect repair in a rat model
Source: J Transl Med. 2018 May 22;16:137. doi: 10.1186/s12967-018-1517-4 (PMC5964689; doi:10.1186/s12967-018-1517-4)
Supplement: Supplementary file 3 — Additional file 3: Table S1. BV/TV (%) of the micro-CT evaluation. Table S2. p value of comparison of BV/TV. Table S3. Area of fluorochromes stained bone (%). Table S4. p value of comparison of fluorochromes stained area. [file 12967_2018_1517_MOESM3_ESM.pdf]

Table S1

BV/TV (%) of the micro-CT evaluation

	non-irradiated		irradiated	
	4W	8W	4W	8W
+EPC	21.77±0.91	28.82±2.84	36.59±2.59	42.11±1.46
BMSC	23.40±1.25	27.61±2.23	20.7±1.78	29.28±2.58
CTR	21.82±2.41	25.49±2.14	17.57±2.81	24.65±1.57

Results are expressed as mean ± SD and n = 4 per group

Table S2

P value of comparison of BV/TV

	non-irradiated		irradiated	
	4W	8W	4W	8W
+EPC vs. BMSC vs. CTR[a]	0.330715	0.200221	0.000003	0.000001
+EPC vs. BMSC[b]	0.383129	0.766459	0.000019	0.000016
+EPC vs. CTR[b]	0.998692	0.182569	0.000004	0.000001
BMSC vs. CTR[b]	0.406618	0.462653	0.203214	0.019819

[a] Data are analyzed by the one way ANOVA

[b] Data are analyzed by tukey post-test

Table S3

Area of fluorochromes stained bone (%)

	non-irradiated			Irradiated		
	AL	CA	TE	AL	CA	TE
+EPC	12.89±0.79	18.93±2.04	12.20±1.07	2.06±0.19	25.21±2.50	17.82±1.44
BMSC	13.03±1.38	21.34±1.34	14.36±1.34	2.04±0.15	13.18±0.49	15.57±1.65
CTR	4.14±0.71	15.20±1.83	12.56±1.16	1.65±0.31	8.02±0.68	5.49±0.77

Results are expressed as mean ± SD and n = 3 per group

Alizarin Red S (AL), Calcein (CA), Tetracycline Hydrochloride (TE)

Table S4

P value of comparison of fluorochromes stained area

	non-irradiated			Irradiated		
	AL	CA	TE	AL	CA	TE
+EPC vs. BMSC vs. CTR[a]	0.000052	0.058147	0.137048	0.113648	0.000024	0.000065
+EPC vs. BMSC[b]	0.984276	0.495389	0.146907	0.991185	0.000171	0.179262
+EPC vs. CTR[b]	0.000097	0.230939	0.929615	0.140565	0.000022	0.000073
BMSC vs. CTR[b]	0.000089	0.050717	0.233282	0.165215	0.014131	0.000231

[a] Data are analyzed by the one way ANOVA

[b] Data are analyzed by tukey post-test

Alizarin Red S (AL), Calcein (CA), Tetracycline Hydrochloride (TE)
